# Effects of exercise on bone mineral density and bone turnover markers in adults: a systematic review and meta-analysis

**DOI:** 10.3389/fphys.2025.1672997

**Published:** 2026-01-08

**Authors:** Tingting Miao, Xun Li, Wenhua Zhang, Jie Liu, Yi Xiao, Xiaoqiang Wang

**Affiliations:** 1 School of Graduate Education, Shandong Sport University, Jinan, Shandong, China; 2 School of Sport and Health, Shandong Sport University, Jinan, Shandong, China; 3 College of Physical Education and Health, Guangxi Normal University, Guilin, Guangxi, China

**Keywords:** exercise, BMD, bone turnover markers, adults, meta-analysis

## Abstract

**Background:**

The incidence of osteoporosis and associated fracture risk increases significantly with age, making it a major global public health concern.

**Objective:**

This study aims to evaluate the impact of exercise on bone mineral density (BMD) at the lumbar spine, femoral neck, and total body in adult women across different age groups, and to further assess the efficacy of different exercise modalities on BMD at these sites. A parallel objective is to investigate the effects of exercise on key bone turnover markers, including the bone formation markers osteocalcin (OC), bone-specific alkaline phosphatase (BALP), and procollagen type I N-terminal propeptide (P1NP), as well as the bone resorption markers C-terminal telopeptide of type I collagen (CTX) and tartrate-resistant acid phosphatase 5b (TRACP-5b). The ultimate goal is to provide evidence for optimizing exercise strategies to enhance bone mass and prevent osteoporosis.

**Methods:**

A systematic search was conducted in PubMed, Embase, the Cochrane Library, and Web of Science from inception to 14 November 2025. Randomized controlled trials (RCTs) were screened according to predefined inclusion and exclusion criteria. Relevant data were extracted, and study quality was assessed using the cochrane risk of bias tool (ROB2). Meta-analyses were performed using Stata 17. Publication bias was evaluated using Egger’s test, and sensitivity analyses were conducted to assess the robustness of the findings.

**Results:**

A total of 22 RCTs involving 1,051 participants were included. The meta-analysis showed that, in subgroup analyses by age, exercise significantly increased lumbar spine BMD [SMD = 0.46, 95% CI (0.16, 0.76), P < 0.01] and femoral neck BMD [SMD = 0.42, 95% CI (0.13, 0.71), P < 0.01] in young adult women under 30 years of age. Subgroup analysis by exercise modality indicated that combined exercise significantly improved femoral neck BMD [SMD = 0.49, 95% CI (0.08, 0.90), P = 0.02] and total body BMD [SMD = 0.52, 95% CI (0.08, 0.97), P = 0.02]. Furthermore, exercise significantly elevated levels of bone formation markers, including OC [SMD = 0.41, 95% CI (0.17, 0.64), P < 0.01] and BALP [SMD = 0.71, 95% CI (0.36, 1.06), P < 0.01]. Subgroup analysis by exercise session duration showed that exercise programs shorter than 4 months were associated with increased OC [SMD = 0.41, 95% CI (0.12, 0.71), P < 0.01] and P1NP [SMD = 0.69, 95% CI (0.14, 1.24), P = 0.01], while BALP levels were significantly elevated both in interventions shorter than 4 months [SMD = 0.56, 95% CI (0.10, 1.01), P < 0.01] and those longer than 4 months [SMD = 0.94,95% CI (0.39, 1.48), P = 0.02].

**Conclusion:**

This systematic review and meta-analysis indicate that exercise significantly increases lumbar spine and femoral neck BMD in young adult women under the age of 30. Additionally, combined exercise shows significant benefits in improving femoral neck and whole-body BMD in adult women. Furthermore, in terms of bone metabolism, exercise effectively promotes the elevation of bone formation markers OC and BALP in adults. Specifically, short-term interventions (less than 4 months) significantly increase OC and P1NP levels, while BALP levels show significant increases following both short-term and long-term (≥4 months) interventions.

**Systematic Review Registration:**

https://www.crd.york.ac.uk/PROSPERO/, identifier CRD420251001516.

## 1 Introduction

Osteoporosis is a systemic skeletal disorder characterized by reduced bone mass and the deterioration of bone microarchitecture, leading to decreased bone strength and increased bone fragility, which significantly elevates the risk of fractures (Johnston and Dagar, 2020). According to the NICE clinical guidelines, the prevalence of osteoporosis increases significantly with age, rising from 2% in women aged 50 to over 25% in those aged 80 (NICE, 2017). Globally, the prevalence of osteoporosis is estimated at 18.3%, with particularly high rates among older adults in Asia, reaching 24.3% (Hsu et al., 2024). Currently, an estimated 137 million women and 21 million men worldwide are at risk of osteoporotic fractures (Odén et al., 2015). Moreover, osteoporosis imposes a substantial economic burden worldwide. In the European Union alone, the cost of prevention and management reached €37 billion in 2010, with projections suggesting a 25% increase by 2025 (Li et al., 2024). Consequently, osteoporosis has emerged as a major global public health challenge.

The development of osteoporosis is primarily attributed to an imbalance between osteoblast and osteoclast activity, typically manifesting as increased bone resorption coupled with reduced bone formation. Mechanical loading through exercise not only enhances bone formation by increasing the flow of extracellular fluid and hydrostatic pressure within the bone marrow—thereby stimulating the proliferation and differentiation of osteoprogenitor cells and promoting osteoblast activation—but also reduces bone resorption. Specifically, mechanical strain decreases the expression of receptor activator of nuclear factor κB ligand (RANKL), limiting its interaction with receptors (RANK) on osteoclast precursors and ultimately reducing osteoclast activity (Muñoz et al., 2020). As a dynamic tissue, bone can adapt to mechanical loading through structural and functional modifications, including changes in bone mass, geometry, and strength (Mustafy et al., 2019; Hou et al., 2024). These mechanical loads arise from both external factors, such as ground reaction forces and inertial forces, and internal factors, such as joint contact forces and muscle contractions.

Throughout the human lifespan, bone undergoes continuous growth during the first 2 decades, reaching peak bone mass around the age of 30, followed by gradual bone loss, with an accelerated decline typically observed after the age of 50 (Hendrickx et al., 2015; Jaividhya and Hallie, 2018). A study by Florence et al. (2023) demonstrated that jumping exercises are less effective in improving bone mineral density (BMD) in individuals over 50 years of age compared to younger adults. This diminished response may be related to age-associated impairments in osteoblast differentiation signaling and degeneration of the lacunar–canalicular network, which weakens the ability of osteocytes to sense and respond to mechanical loading (Chermside-Scabbo et al., 2020). Consequently, regular exercise during adulthood plays a critical role in optimizing peak bone mass, maintaining bone homeostasis, and preventing age-related bone loss and osteoporosis in later life.

However, most previous research has focused on the effects of exercise on BMD in postmenopausal women (Watson et al., 2018; Kemmler et al., 2020; Mohebbi et al., 2023). This meta-analysis primarily focuses on premenopausal women, systematically comparing the effects of exercise on BMD in different age groups within this population, specifically examining the lumbar spine, femoral neck, and whole-body BMD. It further explores the differential effects of various exercise modalities on BMD across different sites, aiming to provide evidence-based recommendations for maintaining and improving bone mass in adult women, as well as for the early prevention of postmenopausal osteoporosis. Additionally, to better understand the underlying physiological mechanisms by which exercise impacts bone health, this study also examines the effects of exercise on bone metabolism biomarkers in adults. These biomarkers include bone formation markers, such as osteocalcin (OC), type I collagen N-terminal propeptide (P1NP), and bone-specific alkaline phosphatase (BALP), as well as bone resorption markers, including type I collagen C-terminal telopeptide (CTX) and tartrate-resistant acid phosphatase 5b (TRACP-5b). Furthermore, we compare the effects of different intervention periods on these bone metabolism markers. Through these analyses, this study aims to provide important scientific evidence to support exercise interventions for bone health.

## 2 Methods

### 2.1 Search strategy

This systematic review and meta-analysis was conducted in accordance with the Preferred Reporting Items for Systematic Reviews and Meta-Analyses (PRISMA) guidelines (Moher et al., 2009). It has been prospectively registered with the International Prospective Register of Systematic Reviews (PROSPERO) under registration number CRD420251001516.

Literature searches were conducted in the following databases: PubMed, Embase, The Cochrane Library, and Web of Science from database inception to 14 November 2025. For example, the PubMed search combined keywords and MeSH terms, including: (((“Adult” [Mesh]) OR “Young Adult” [Mesh]) AND ((((“Exercise” [Mesh]) OR “Plyometric Exercise” [Mesh]) OR “Resistance Training” [Mesh]) OR ((((aerobics) OR (physical activity)) OR (training)) OR (sport)))) AND ((((((bone) OR (bone health)) OR (Bone mineral density)) OR (bone metabolism)) OR (bone turnover)) OR (bone biomarkers)) Filters: Randomized Controlled Trial.

### 2.2 Inclusion and exclusion criteria

Studies were eligible if they met the following criteria: (a) Study design: randomized controlled trials (RCTs); (b) Participants: adults aged 18–45 years; (c) Intervention: any form of long-term exercise; (d) Control: routine daily activities or regular exercise without additional interventions (e) Outcomes: at least one site of BMD (e.g., lumbar spine, femoral neck or whole-body); or at least one type of bone turnover marker (e.g., OC, BALP, P1NP, CTX, or TRACP-5b).

Exclusion criteria were: (a) Non-randomized controlled trials; (b) Participants under 18 years of age or over 45 years of age (particularly postmenopausal women), as well as those with comorbidities that affect bone metabolism (e.g., hyperparathyroidism, osteogenesis imperfecta); (c) Control groups not meeting the criteria (e.g., no control group, medication-based controls); (d) Studies lacking BMD or bone turnover marker outcomes; (e) Insufficient or unclear data reporting, preventing calculation of means and standard deviations; (f) Acute studies or interventions with a duration of less than 8 weeks; (g) Follow-up studies, reviews, case reports, editorials, conference abstracts, and letters; (h) Animal studies.

### 2.3 Data extraction

Miao screened the titles and abstracts to exclude irrelevant studies. Zhang, Liu and Xiao independently reviewed the full texts and extracted data from the eligible studies; any disagreements were resolved through discussion with Miao. Extracted data included: (a) Basic characteristics: authors, publication year, sample size and age of participants in the exercise group and control group; (b) Exercise characteristics: type of exercise, exercise session duration, frequency, intervention period, exercise intensity, and outcome measures for BMD and bone turnover markers. For quantitative outcomes, post-intervention means and standard deviations were extracted.

### 2.4 Risk of bias assessment

Two independent reviewers (Zhang and Liu) used the revised Cochrane risk-of-bias tool for randomized trials (ROB 2) to assess the risk of bias of included studies from five domains: the randomization process, deviations from intended interventions, missing outcome data, measurement of the outcome, and selection of the reported result. Each domain was judged as “low risk,” “some concerns,” or “high risk” according to corresponding algorithms. After learning the Cochrane risk-of-bias tool and pre-assessed, two independent reviewers assessed the risk of bias and then cross-checked. Two reviewers discussed the disagreements or consulted with a third reviewer (Xiao).

### 2.5 Data analysis

The outcome measures of the included studies were analyzed using Stata17. The outcome indicators of the studies included in this analysis were all continuous variables, standardized mean differences (SMDs) with 95% confidence intervals (95% CIs) were selected as the effect measures for pooling the effect sizes. According to the Cochrane Handbook (Chapter 10.10.2), the degree of heterogeneity is interpreted as follows: 0%–40% may not be of practical significance; 30%–60% suggests moderate heterogeneity; 50%–90% indicates high heterogeneity; and 75%–100% suggests very high heterogeneity (Cochrane Collaboration, 2024). If I^2^ ≥ 50%, a random-effects model was applied, and subgroup analysis was conducted to explore the sources of heterogeneity. Sensitivity analysis was also performed to ensure the reliability of the results. When more than 10 studies were included, Egger’s test was used to assess publication bias. The statistical significance level for all results was set at P < 0.05.

## 3 Results

### 3.1 Search results

**FIGURE 1:**
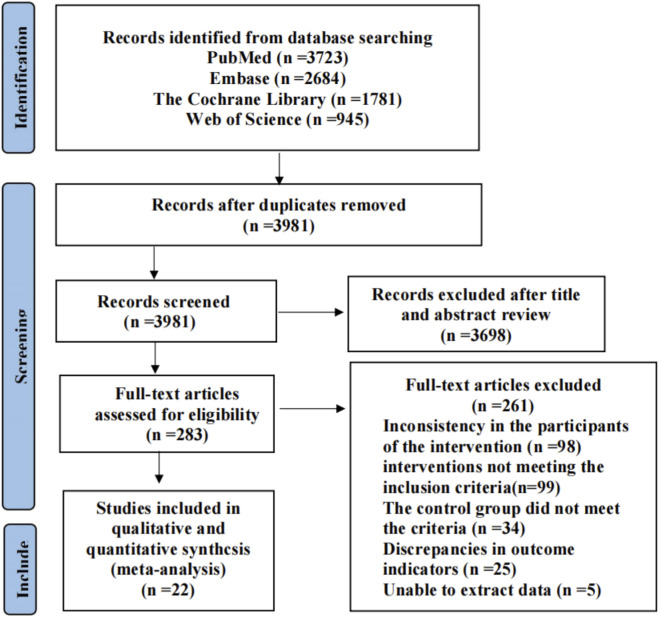
PRISMA study flow diagram.

A total of 9,133 RCTs were initially identified, including 3,723 from PubMed, 2,684 from Embase, 1,781 from The Cochrane Library, and 945 from Web of Science. These records were imported into EndNote, and after removing duplicates, 3,981 studies remained. After screening titles and abstracts, 3,698 studies were excluded, leaving 283 studies for full-text review. Of these, studies were excluded due to inconsistency in the target population of the intervention (n = 98), interventions not meeting the inclusion criteria (n = 99), control group not meeting the eligibility criteria (n = 34), discrepancies in outcome indicators (n = 25), and inability to extract data (n = 5). Ultimately, 22 studies were included in the meta-analysis (Friedlander et al., 1995; Lohman et al., 1995; Bassey et al., 1998; Torvinen et al., 2003; Schroeder et al., 2004; Vainionpää et al., 2005; Kato et al., 2006; Heikkinen et al., 2007; Warren et al., 2008; Guadalupe-Grau et al., 2009; Humphries et al., 2009; Lovelady et al., 2009; Vainionpää et al., 2009; Krustrup et al., 2010; Colleran et al., 2012; Wang et al., 2012; Mosti et al., 2014; Kim et al., 2015; Ploutz-Snyder et al., 2018; Hornstrup et al., 2019; Batrakoulis et al., 2021; Kobayashi et al., 2023). Study flowchart shown in Figure 1.

### 3.2 Basic characteristics of the included studies

A total of 22 RCTs published between 1995 (Friedlander et al., 1995) and 2023 (Kobayashi et al., 2023) were included, involving 1,051 participants, with 547 in the exercise groups and 504 in the control groups. The sample size in the exercise groups ranged from 6 (Kobayashi et al., 2023) to 72 (Warren et al., 2008) participants per study. Participants’ ages ranged from approximately 19 (Kobayashi et al., 2023) to 43 (Krustrup et al., 2010) years. Among the included studies, 15 focused primarily on female participants, all of whom were premenopausal women (Friedlander et al., 1995; Lohman et al., 1995; Bassey et al., 1998; Schroeder et al., 2004; Vainionpää et al., 2005; Kato et al., 2006; Heikkinen et al., 2007; Warren et al., 2008; Humphries et al., 2009; Lovelady et al., 2009; Vainionpää et al., 2009; Krustrup et al., 2010; Colleran et al., 2012; Mosti et al., 2014; Batrakoulis et al., 2021; Kobayashi et al., 2023); three studies focused primarily on male participants (Wang et al., 2012; Kim et al., 2015; Hornstrup et al., 2019); and the remaining three included both male and female participants (Torvinen et al., 2003; Guadalupe-Grau et al., 2009; Ploutz-Snyder et al., 2018). Baseline characteristics are shown in Table 1.

**TABLE 1:** Basic characteristics of the included studies.

Author (year)	Sample size (n)		Mean age (years)		Sex
	EX	C	EX	C	
Bassey et al. (1998)	30	25	38.4 ± 7.4	36.4 ± 7.6	Women
Batrakoulis et al. (2021)	14	21	36.4 ± 5.0	36.0 ± 4.2	Women
Colleran et al. (2012)	14	13	31.9 ± 3.1	30.3 ± 3.8	Women
Friedlander et al. (1995)	32	31	28.0 ± 6.8	30.1 ± 4.0	Women
Guadalupe-Grau et al. (2009)	Women: 8 Men: 16	Women: 15 Men: 27	Women: 22.3 ± 2.7 Men: 23.13 ± 2.2	Women: 23.7 ± 2.7 Men: 24.6 ± 2.4	Women and men
Heikkinen et al. (2007)	34	30	38.3 ± 1.9	38.3 ± 1.6	Women
Hornstrup et al. (2019)	14	12	24.2 ± 2.8	25.8 ± 2.8	Men
Humphries et al. (2009)	WBV: 15 WBV + RT: 15	15	21.02 ± 3.39	21.02 ± 3.39	Women
Kato et al. (2006)	18	18	20.5 ± 0.6	20.9 ± 0.8	Women
Kim et al. (2015)	29	10	24.86 ± 2.75	26.60 ± 2.80	Men
Kobayashi et al. (2023)	RRT: 6 NRT: 8	RCON: 8 NCON: 6	RRT: 20.0 ± 0.9 NRT: 20.0 ± 1.1	RCON: 19.6 ± 2.0 NCON: 21.2 ± 2.0	Women
Krustrup et al. (2010)	FG: 9 RG: 10	9	40 ± 3	40 ± 3	Women
Lohman et al. (1995)	22	34	34.2 ± 2.6	34.4 ± 2.7	Women
Lovelady et al. (2009)	10	10	31.1 ± 1.0	31.6 ± 0.9	Women
Mosti et al. (2014)	14	15	22.7 ± 2.2	21.5 ± 2.2	Women
Torvinen et al. (2003)	27	26	23.1 ± 4.3	25.5 ± 5.8	Women and men
Schroeder et al. (2004)	LRT: 14 HRT: 14	9	LRT: 24.4 ± 1.9 HRT: 24.0 ± 1.4	24.4 ± 2.2	Women
Ploutz-Snyder et al. (2018)	EX1: 9 EX2: 8	9	EX1: 33 ± 10 EX2: 29 ± 5	37 ± 8	Women and men
Vainionpää et al. (2005)	39	41	38.1 ± 1.7	38.5 ± 1.6	Women
Vainionpää et al. (2009)	39	41	38.1 ± 1.9	38.2 ± 1.6	Women
Wang et al. (2012)	7	7	30.7 ± 4.6	30.1 ± 3.1	Men
Warren et al. (2008)	72	72	36.4 ± 5.5	36.2 ± 5.6	Women

EX, exercise; C, group control group; WBV, whole-body vibration; RT, resistance training; RRT, runner with resistance training group; NRT, non-athlete with resistance training group; RCON, runner control group; NCON, non-athlete control group; FG, football group; RG, running group; LRT, low-intensity resistance training; HRT, high-intensity resistance training; EX1, training using traditional equipment; EX2, training using flywheel device.

### 3.3 Characteristics of the exercise interventions included in the study

The exercise category included in this study primarily consisted of high-impact exercises, resistance training, combined exercise, and whole-body vibration training. The types of exercise covered a range of activities, including jumping, small-ball games (e.g., soccer, handball), circuit training, resistance exercises using equipment, and vibration platform training. The duration of each session varied significantly, ranging from very short sessions of a few minutes (e.g., 4-min vibration training (Torvinen et al., 2003), <2-min jumping exercises (Kato et al., 2006)) to longer sessions lasting up to 60 min (Friedlander et al., 1995; Lohman et al., 1995; Vainionpää et al., 2005; Heikkinen et al., 2007; Vainionpää et al., 2009; Krustrup et al., 2010; Ploutz-Snyder et al., 2018; Kobayashi et al., 2023). The frequency of exercise was generally between 2 and 4 times per week, with the intervention periods spanning a wide range, from 2 months (Guadalupe-Grau et al., 2009; Wang et al., 2012; Kim et al., 2015; Ploutz-Snyder et al., 2018) to 2 years (Friedlander et al., 1995; Warren et al., 2008). Studies with intervention periods shorter than 4 months (Guadalupe-Grau et al., 2009; Wang et al., 2012; Mosti et al., 2014; Kim et al., 2015; Ploutz-Snyder et al., 2018; Hornstrup et al., 2019) primarily focused on examining the effects of exercise on bone metabolism.

The intensity of the exercises was specifically tailored for each modality. High-impact exercises were quantified based on ground reaction forces (up to 3–4 times body weight) and accelerations (ranging from 2 to 9 g). Resistance training typically involved moderate to high loads (e.g., 60%–90% of 1RM) and emphasized concentric phase acceleration. Aerobic exercises were usually performed at 65%–85% of maximum heart rate or VO_2_max. Combined exercise protocols integrated various elements of these intensities. The primary outcome measures included DXA-assessed BMD at the whole-body, lumbar spine, and femoral neck sites, along with serum biomarkers of bone formation (e.g., P1NP, OC, BALP) and bone resorption (e.g., CTX, TRACP-5b). Exercise characteristics are summarized in Table 2.

**TABLE 2:** Characteristics of the exercise interventions included in the study.

Author (year)	Exercise category	Exercise type	Exercise duration	Frequency	Intervention period	Exercise intensity	Outcome indicators
Bassey et al. (1998)	High-impact exercise	Vertical jump	10 min	6 times/week	5 months	Jump count: 50/day; Jump height: avg 8.5 cm; Ground reaction force: ∼3–4x body weight; Frequency: 1 Hz	LS, FN
Batrakoulis et al. (2021)	Combined exercise	RT: using kettlebells, medicine balls; AT: battle ropes, speed ladder; Balance and joint mobility training	23–41 min	3 times/week	10 months	Heart rate: ≥75% max HR	WB
Colleran et al. (2012)	Combined exercise	AT: Walking; RT: Squats, bench press, etc; High-impact exercise: 50 vertical jumps from the 9th week	AT: Not specified RT:20–30 min	AT: >5 days/week, RT: 3 days/week	4 months	AT: Moderate intensity (steps ≥100/min) RT: Progressive load, specific intensity not specified	WB, LS
Friedlander et al. (1995)	Combined exercise	AT: Running; RT: Dumbbells, barbells, etc.	60 min	3 times/week	2 years	AT: 70%–85% max HR; RT: Light-to-moderate load (3–12 lbs dumbbells, 16–36 lbs barbells), gradually increasing load	LS, FN
Guadalupe-Grau et al. (2009)	Combined exercise	Jump training: Drop jumps, hurdle jumps; RT: Leg extensions	Not specified	3 times/week	2 months	RT: 50%–90% 1RM	OC
Heikkinen et al. (2007)	High-impact exercise	Jumping, Running	60 min	3 times/week	12 months	Acceleration range: Running (9 km/h): ∼1,000 m/s^3^, Running (13 km/h): ∼1,500 m/s^3^, jumping: ∼2,000–3,500 m/s^3^	LS, FN
Hornstrup et al. (2019)	Combined exercise	Handball training	40 min	1.4–2.8 times/week	3 months	Avg HR: 84% ± 4% max HR	OC, P1NP, CTX
Humphries et al. (2009)	(1) WBV (2) WBV + RT	(1) WBV (2) WBV + RT (Smith machine squats)	Whole-body Vibration: 3 min	2 times/week	4 months	(1) Frequency: 50 Hz, amplitude: 1–6 mm, (2) WBV + RT: 75% body weight (1–10 reps), 100% body weight (11–20 reps), 125% body weight (21–32 reps)	LS, FN, BALP
Kato et al. (2006)	High-impact exercise	Jumping	<2 min	3 times/week	6 months	Ground reaction force: Take-off: 2.35 ± 0.25× body weight, landing: 4.76 ± 0.86× body weight	LS, FN
Kim et al. (2015)	AT	Treadmill running	Not specified	4 times/week	2 months	65%–75% VO_2_max	OC, BALP
Kobayashi et al. (2023)	RT	Squats and deadlifts	60 min	2 times/week	4 months	60%–85% 1RM	WB, LS, FN, P1NP
Krustrup et al. (2010)	(1) Combined exercise (2) AT	(1) FG: Small football games (2) RG: Outdoor continuous running	60 min	1.9–1.7 times/week	16 months	(1) Avg HR: 82% → 81% max HR; (2) Avg HR: 81%–82% max HR	WB
Lohman et al. (1995)	RT	Bench press, leg curls, etc.	60 min	3 times/week	18 months	70% 1RM initially, 75% after 6 months, 80% after 12 months	LS, FN, WB
Lovelady et al. (2009)	Combined exercise	AT: Brisk walking RT: Squats, bench press, etc.	45 min	6 times/week	4 months	AT: Target HR 65%–80% max HR; RT: Progressively increasing load	WB, LS
Mosti et al. (2014)	RT	Hack squat	20 min	3 times/week	3 months	85%–90% 1RM, emphasizing concentric phase max acceleration	P1NP, CTX
Torvinen et al. (2003)	WBV	Light squats (0–10 s), Standing (10–20 s)	4 min	3–5 times/week	8 months	Frequency: 25–45 Hz; Vertical acceleration: 2–8 g Amplitude: 2 mm	OC, PINP CTX, TRACP-5b
Schroeder et al. (2004)	RT	Chest press, high-pulley pull-down	40 min	2 times/week	4 months	HRT: 125% concentric 1RM, 3 sets × 6 reps LRT: 75% concentric 1RM, 3 sets × 10 reps	WB, LS, OC
Ploutz-Snyder et al. (2018)	(1) EX1: Combined exercise (2) EX2: Combined exercise	(1) EX1: AT + RT (2) EX2: AT + RT	60 min	Aerobic: 6 times/week, Resistance: 3 times/week	2 months	AT: Interval training: 85%–96% max HR RT: Non-linear periodization, weekly load increase	OC, BALP
Vainionpää et al. (2005)	High-impact exercise	Jumping, Running	60 min	3 times/week	12 months	Gradually increasing intensity and impact	LS, FN
Vainionpää et al. (2009)	High-impact exercise	Jumping, Running	60 min	3 times/week	12 months	Using accelerometer to quantify impact intensity, divided into acceleration levels: 2.5–5.3 g (running, jumping), 5.4–9.2 g (high-impact jumping)	PINP, TRACP-5b
Wang et al. (2012)	Combined exercise	Vertical vibration platform (with resistance bands for waist and shoulders)	24 min	7 times/week	2 months	Vibration frequency: 30 Hz, acceleration: 0.3 g, displacement: <0.1 mm, Resistance: 1.5× body weight	OC, BALP, TRACP-5b
Warren et al. (2008)	RT	Bench press, leg press, etc.	Not specified	2 times/week	2 years	Low-intensity training for first 3 weeks, then load adjusted to complete 3 sets of 10 reps	LS, FN

AT, aerobic training; RT, resistance training; WBV, whole-body vibration; WB, whole-body; LS, lumbar spine; FN, femoral neck; OC, osteocalcin; CTX, C-terminal telopeptide of type I collagen; BALP, bone-specific alkaline phosphatase; TRACP-5b, tartrate-resistant acid phosphatase 5b; FG, football group; RG, running group; LRT, low-intensity resistance training; HRT, high-intensity resistance training; EX1, training using traditional equipment; EX2, training using flywheel device.

### 3.4 Risk of bias

**FIGURE 2:**
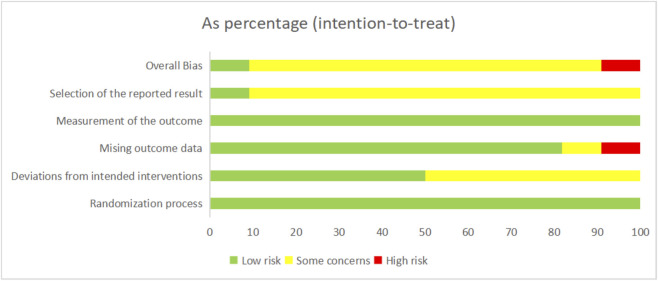
Risk of bias of the included studies.

**FIGURE 3:**
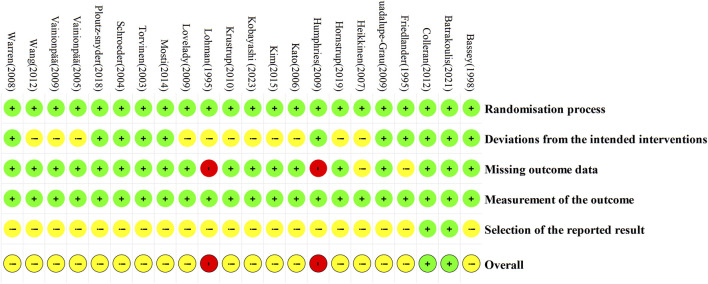
Risk of bias summary of the included studies.

All studies were randomized, with two studies providing specific information on allocation concealment (Friedlander et al., 1995; Colleran et al., 2012). Eleven studies used intention-to-treat analysis (Friedlander et al., 1995; Bassey et al., 1998; Torvinen et al., 2003; Schroeder et al., 2004; Warren et al., 2008; Guadalupe-Grau et al., 2009; Humphries et al., 2009; Colleran et al., 2012; Mosti et al., 2014; Ploutz-Snyder et al., 2018; Batrakoulis et al., 2021), while the remaining studies employed case analysis. Four studies had a high dropout rate, and were therefore rated as having some concerns or high risk (Friedlander et al., 1995; Lohman et al., 1995; Heikkinen et al., 2007; Humphries et al., 2009). The primary outcome measures were all objectively assessed, minimizing the potential for subjective bias. Two studies provided registration numbers (Colleran et al., 2012; Batrakoulis et al., 2021). Overall, two studies were rated as low risk (Colleran et al., 2012; Batrakoulis et al., 2021), two as high risk (Lohman et al., 1995; Humphries et al., 2009), and the remaining studies were rated as having some concerns (Figures 2, 3).

### 3.5 Meta-analysis results

#### 3.5.1 Effect of exercise on lumbar spine bone mineral density in adult women

**FIGURE 4:**
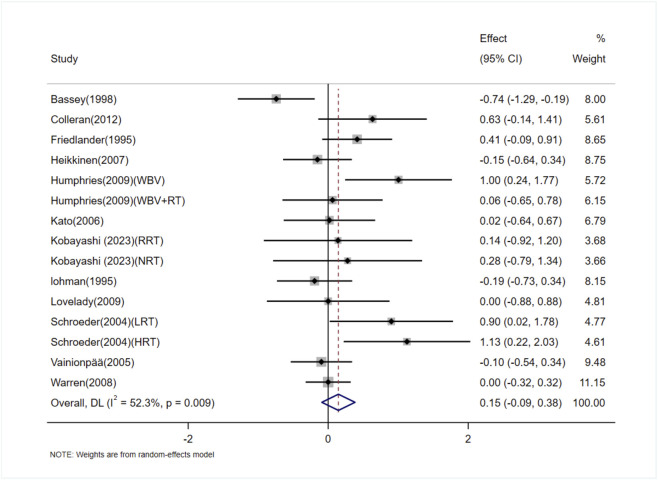
Forest plot of the meta-analysis on the effects of exercise on lumbar spine BMD. WBV, whole-body vibration; RT, resistance training; RRT, runner with resistance training group; NRT, non-athlete with resistance training group; LRT, low-intensity resistance training; HRT, high-intensity resistance training.

The effect of exercise on lumbar spine BMD in adult women was assessed in 12 studies (15 trials) (Friedlander et al., 1995; Lohman et al., 1995; Bassey et al., 1998; Schroeder et al., 2004; Vainionpää et al., 2005; Kato et al., 2006; Heikkinen et al., 2007; Warren et al., 2008; Humphries et al., 2009; Lovelady et al., 2009; Colleran et al., 2012; Kobayashi et al., 2023). The heterogeneity between studies was moderate (I^2^ = 52.3%), and a random-effects model was applied for the analysis. The results indicated no significant difference in lumbar spine BMD between the exercise and control groups [SMD = 0.15, 95% CI (−0.09, 0.38), P = 0.23] (Figure 4). The Egger test suggested no significant publication bias (P = 0.08).

##### 3.5.1.1 Subgroup analysis

Subgroup analyses based on age and exercise modality were conducted to further investigate the effects on lumbar spine BMD in adult women. As shown in Table 3.

**TABLE 3:** Subgroup analyses of the effects of exercise on lumbar spine BMD.

Subgroup	Category	Number of trials (n)	SMD (95% CI)	P-value
Age	>30 years	7	−0.12 (−0.36, 0.13)	0.35
	<30 years	8	0.46 (0.16, 0.76)	<0.01
Exercise category	High impact	4	−0.24 (−0.56, 0.07)	0.13
	Combined exercise	4	0.32 (−0.02, 0.65)	0.06
	WBV	1	1 (0.24, 1.77)	0.01
	RT	4	0.27 (−0.14, 0.67)	0.19

RT, resistance training; WBV, whole-body vibration.

#### 3.5.2 Effect of exercise on femoral neck bone mineral density in adult women

**FIGURE 5:**
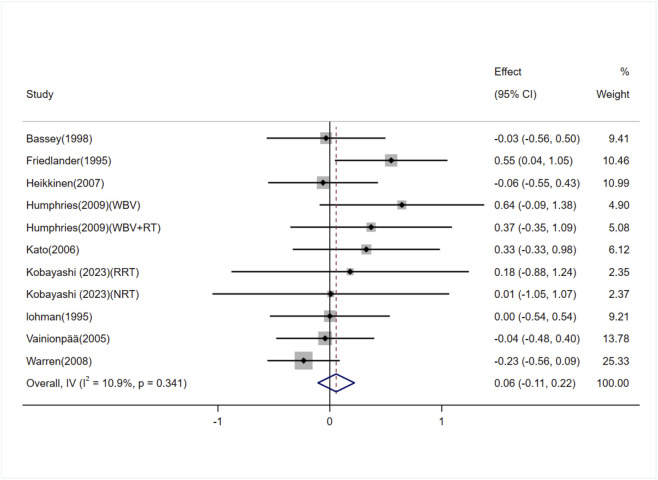
Forest plot of the meta-analysis on the effects of exercise on femoral neck BMD. WBV, whole-body vibration; RT, resistance training; RRT, runner with resistance training group; NRT, non-athlete with resistance training group.

The effect of exercise on femoral neck BMD in adult women was evaluated in nine studies (11 trials) (Friedlander et al., 1995; Lohman et al., 1995; Bassey et al., 1998; Vainionpää et al., 2005; Kato et al., 2006; Heikkinen et al., 2007; Warren et al., 2008; Humphries et al., 2009; Kobayashi et al., 2023). The heterogeneity between studies was low (I^2^ = 10.9%), and a fixed-effects model was used for the analysis. The results showed no significant difference in femoral neck BMD between the exercise and control groups [SMD = 0.06, 95% CI (−0.11, 0.22), P = 0.49] (Figure 5). The Egger test suggested no significant publication bias (P = 0.07).

##### 3.5.2.1 Subgroup analysis

Subgroup analyses based on age and exercise modality were performed to explore the effects on femoral neck BMD in adult women. As shown in Table 4.

**TABLE 4:** Subgroup analyses of the effects of exercise on femoral neck BMD.

Subgroup	Category	Number of trials (n)	SMD (95% CI)	P-value
Age	>30 years	5	−0.05 (−0.32, 0.23)	0.28
	<30 years	6	0.42 (0.13, 0.71)	<0.01
Exercise category	High impact	4	0.01 (−0.25, 0.27)	0.93
	Combined exercise	2	0.49 (0.08, 0.90)	0.02
	WBV	1	0.64 (−0.09, 1.38)	0.09
	RT	4	−0.14 (−0.40, 0.12)	0.29

RT, resistance training; WBV, whole-body vibration.

#### 3.5.3 Effect of exercise on whole-body bone mineral density in adult women

**FIGURE 6:**
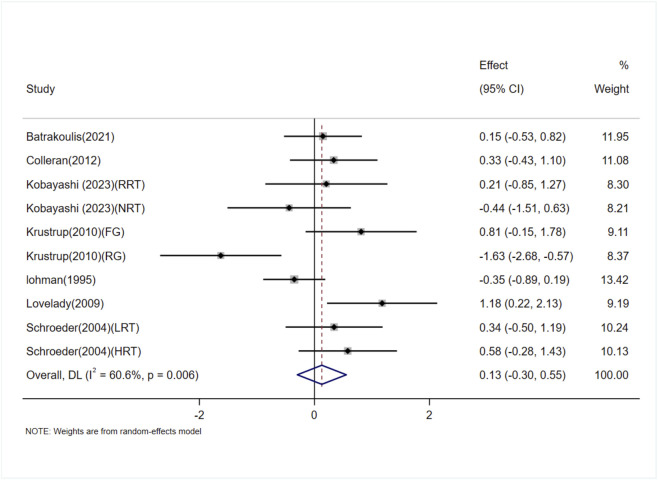
Forest plot of the meta-analysis on the effects of exercise on whole-body BMD.RRT,runner with resistance training group; NRT,non-athlete with resistance training group; FG, football group; RG, running group; LRT,low-intensity resistance training; HRT, high-intensity resistance training.

The effect of exercise on whole-body BMD in adult women was examined in seven studies (10 trials) (Lohman et al., 1995; Schroeder et al., 2004; Lovelady et al., 2009; Krustrup et al., 2010; Colleran et al., 2012; Batrakoulis et al., 2021; Kobayashi et al., 2023). The heterogeneity between studies was high (I^2^ = 60.6%), and a random-effects model was applied. The analysis showed no significant difference in whole-body BMD between the exercise and control groups [SMD = 0.13, 95% CI (−0.30, 0.55), P = 0.56] (Figure 6). The Egger test suggested no significant publication bias (P = 0.71).

##### 3.5.3.1 Subgroup analysis

Subgroup analyses based on age and exercise modality were conducted to assess the impact on whole-body BMD in adult women. As shown in Table 5.

**TABLE 5:** Subgroup analyses of the effects of exercise on whole-body BMD.

Subgroup	Category	Number of trials (n)	SMD (95% CI)	P-value
Age	>30 years	6	0.09 (−0.56, 0.74)	0.79
	<30 years	4	0.24 (−0.23, 0.71)	0.32
Exercise category	RT	5	0.01 (−0.39, 0.41)	0.32
	Combined exercise	4	0.52 (0.08, 0.97)	0.02
	AT	1	−1.63 (−2.68, −0.57)	<0.01

AT, aerobic training; RT, resistance training.

#### 3.5.4 Effects of exercise on OC levels

**FIGURE 7:**
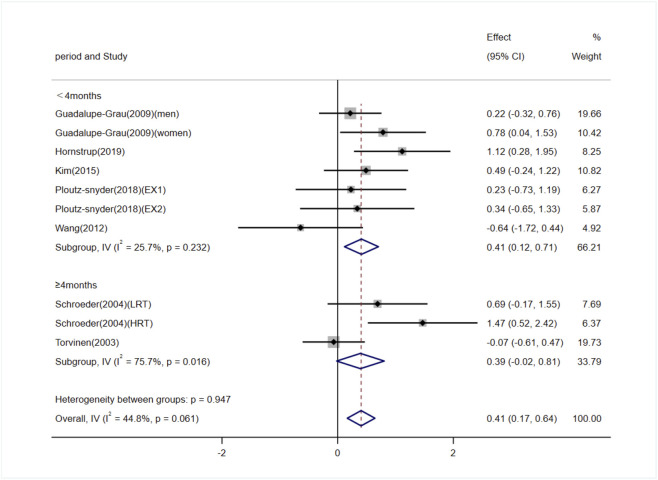
Forest plot of the meta-analysis on the effects of exercise on OC levels. EX1, training using traditional equipment; EX2, training using flywheel device; LRT, low-intensity resistance training; HRT, high-intensity resistance training.

Seven studies (involving 10 trials) investigating the effects of exercise on OC levels in adults (Torvinen et al., 2003; Schroeder et al., 2004; Guadalupe-Grau et al., 2009; Wang et al., 2012; Kim et al., 2015; Ploutz-Snyder et al., 2018; Hornstrup et al., 2019). Among them, five studies (3 trials) reported exercise interventions lasting ≥4 months (Torvinen et al., 2003; Schroeder et al., 2004), and two studies (7 trials) reported interventions lasting <4 months (Guadalupe-Grau et al., 2009; Wang et al., 2012; Kim et al., 2015; Ploutz-Snyder et al., 2018; Hornstrup et al., 2019). The heterogeneity among the included studies was low (I^2^ = 44.8%), so a fixed-effects model was used for meta-analysis. The pooled results showed that exercise significantly increased OC levels compared to the control group [SMD = 0.41, 95% CI (0.17, 0.64), P < 0.01]. Subgroup analysis indicated that exercise interventions lasting ≥4 months showed no significant difference in OC levels compared to the control group [SMD = 0.39, 95% CI (−0.02, 0.81), P = 0.06], whereas interventions lasting <4 months significantly increased OC levels [SMD = 0.41, 95% CI (0.12, 0.71), P < 0.01] (Figure 7). Egger’s test indicated no significant publication bias (P = 0.38).

#### 3.5.5 Effects of exercise on BALP levels

**FIGURE 8:**
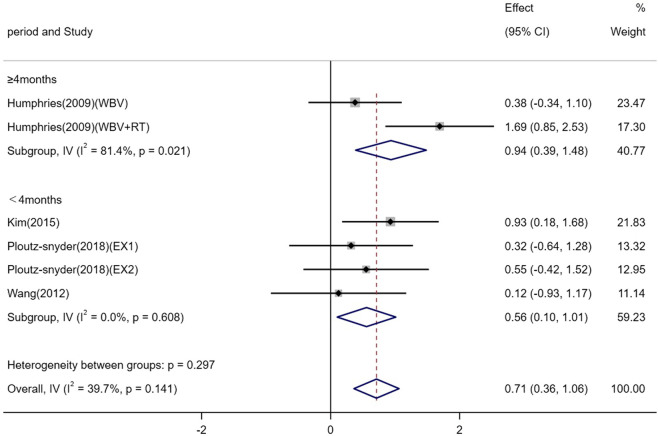
Forest plot of the meta-analysis on the effects of exercise on BALP levels. WBV, whole-body vibration; RT, resistance training; EX1, training using traditional equipment; EX2, training using flywheel device.

Four studies (involving six trials) evaluating the effects of exercise on BALP levels in adults (Humphries et al., 2009; Wang et al., 2012; Kim et al., 2015; Ploutz-Snyder et al., 2018). Among them, one study (2 trials) involved exercise interventions lasting ≥4 months (Humphries et al., 2009), and three studies (4 trials) involved interventions lasting <4 months (Wang et al., 2012; Kim et al., 2015; Ploutz-Snyder et al., 2018). The heterogeneity among the included studies was low (I^2^ = 39.7%), so a fixed-effects model was used for meta-analysis. The pooled results demonstrated that exercise significantly increased BALP levels compared to the control group [SMD = 0.71, 95% CI (0.36, 1.06), P < 0.01]. Subgroup analysis further revealed that both exercise interventions lasting ≥4 months [SMD = 0.94, 95% CI (0.39, 1.48), P = 0.02] and those lasting <4 months [SMD = 0.56, 95% CI (0.10, 1.01), P < 0.01] significantly increased BALP levels (Figure 8).

#### 3.5.6 Effects of exercise on P1NP levels

**FIGURE 9:**
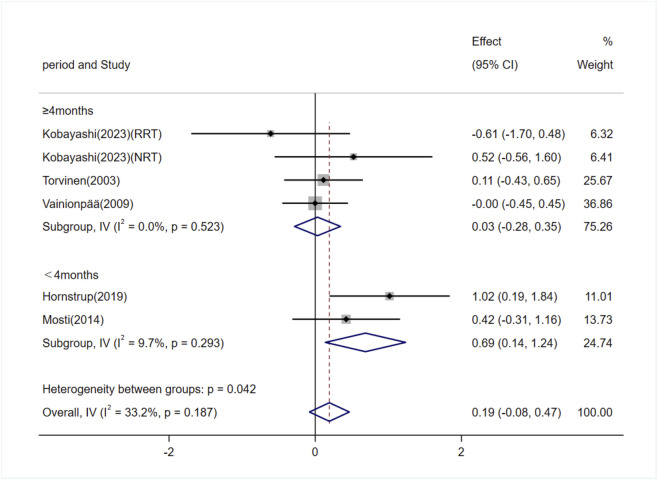
Forest plot of the meta-analysis on the effects of exercise on P1NP levels. RRT, runner with resistance training group; NRT, non-athlete with resistance training group.

Five studies (involving six trials) investigating the effects of exercise on P1NP levels in adults (Torvinen et al., 2003; Vainionpää et al., 2009; Mosti et al., 2014; Hornstrup et al., 2019; Kobayashi et al., 2023). Among them, three studies (4 trials) involved exercise interventions lasting ≥4 months (Torvinen et al., 2003; Vainionpää et al., 2009; Kobayashi et al., 2023), and two studies (2 trials) involved interventions lasting <4 months (Mosti et al., 2014; Hornstrup et al., 2019). The heterogeneity among the included studies was low (I^2^ = 33.2%), so a fixed-effects model was used for meta-analysis. The overall pooled results showed no significant difference in P1NP levels between the exercise and control groups [SMD = 0.19, 95% CI (−0.08, 0.47), P = 0.17]. Subgroup analysis indicated that exercise interventions lasting ≥4 months had no significant effect on P1NP levels [SMD = 0.03, 95% CI (−0.28, 0.35), P = 0.85]. However, interventions lasting <4 months significantly increased P1NP levels [SMD = 0.69, 95% CI (0.14, 1.24), P = 0.01] (Figure 9).

#### 3.5.7 Effects of exercise on CTX levels

**FIGURE 10:**
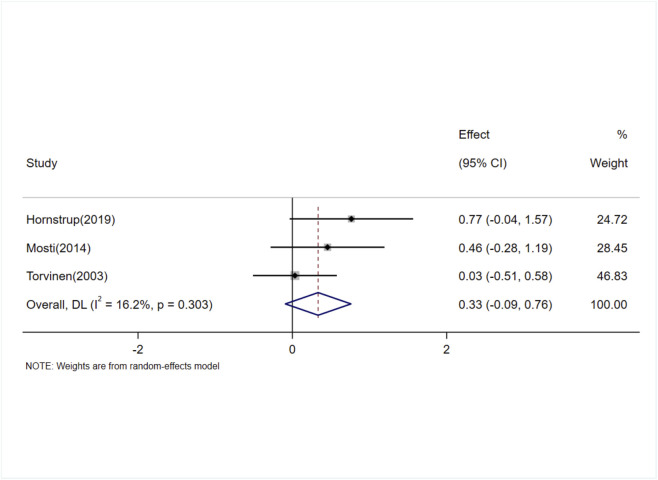
Forest plot of the meta-analysis on the effects of exercise on CTX levels.

Three studies (involving three trials) evaluating the effects of exercise on C-terminal telopeptide of type I collagen (CTX) levels in adults (Torvinen et al., 2003; Mosti et al., 2014; Hornstrup et al., 2019). The heterogeneity among the included studies was low (I^2^ = 16.2%), so a fixed-effects model was used for meta-analysis. The results showed no significant difference in CTX levels between the exercise and control groups [SMD = 0.32, 95% CI (−0.07, 0.70), P = 0.11] (Figure 10).

#### 3.5.8 Effects of exercise on TRACP-5b levels

**FIGURE 11:**
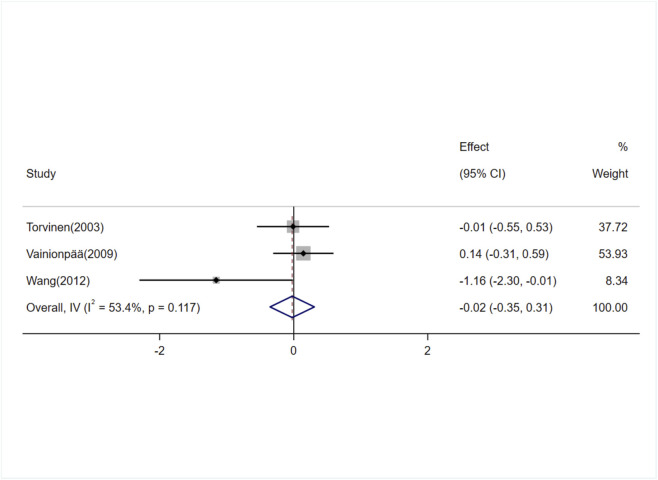
Forest plot of the meta-analysis on the effects of exercise on TRACP-5b levels.

Three studies (involving three trials) investigating the effects of exercise on TRACP-5b levels in adults (Torvinen et al., 2003; Vainionpää et al., 2009; Wang et al., 2012). There was moderate heterogeneity among the included studies (I^2^ = 53.4%), so a random-effects model was used for meta-analysis. The results showed no significant difference in TRACP-5b levels between the exercise and control groups [SMD = −0.13, 95% CI (−0.67, 0.41), P = 0.64] (Figure 11).

### 3.6 Sensitivity analysis

Sensitivity analysis was conducted by sequentially excluding individual studies and recalculating the pooled effect sizes. The results showed that the effect sizes and confidence intervals for all outcomes remained stable, indicating the robustness of the findings (Supplementary Figure S1).

## 4 Discussion

This systematic review and meta-analysis aimed to evaluate the effects of exercise on lumbar spine, femoral neck, and whole-body BMD in adult women. We conducted subgroup analyses based on age to further investigate how women of different age groups respond to exercise interventions. Additionally, the study assessed the effects of different types of exercise on BMD. Furthermore, we provided a comprehensive analysis of exercise’s impact on bone turnover markers in adults, including bone formation markers (OC, BALP, P1NP) and bone resorption markers (CTX, TRACP-5b), with subgroup analyses based on intervention period.

The meta-analysis results demonstrated that exercise significantly improved lumbar spine and femoral neck BMD in young adult women aged under 30. In terms of exercise modalities, combined exercise showed positive effects on enhancing femoral neck and whole-body BMD in adult women. Regarding bone metabolism, exercise significantly increased the levels of bone formation markers OC and BALP. Specifically, short-term exercise (less than 4 months) was more effective in boosting OC and P1NP levels, while BALP levels increased significantly following both short-term and long-term (≥4 months) exercise interventions.

### 4.1 Effects of exercise on bone mineral density in adult women

Maintaining optimal BMD before menopause is crucial for reducing the risk of osteoporosis and related fractures in the future, as fracture risk in this stage can increase by 1.5–3.0 times (Vondracek et al., 2009). Although Kelley et al. (2013) found that exercise had a modest but significant improvement effect on femoral neck (FN) and lumbar spine (LS) BMD in premenopausal women, the overall results of this study did not show a significant improvement in BMD from exercise in premenopausal women. Research has shown that bone mass continues to significantly increase between the ages of 20 and 30 in women after growth ceases, with lumbar spine BMD and total body bone mass increasing by 6.8% and 12.5% per decade, respectively (Recker et al., 1992). Therefore, subgroup analysis in this study was conducted with an average age of 30 years, and the results revealed that exercise significantly improved lumbar spine and femoral neck BMD in premenopausal women under the age of 30. This suggests that exercise interventions during the young adult stage, when bone mass still has growth potential, may have a more positive effect on bone health.

The mechanical load generated by exercise, especially dynamic strains that exceed daily activity levels, can remodel bone tissue, optimize its macro and microstructures, and enhance bone strength (Kohrt et al., 2004). The effects of different types of exercise on bone are site-specific. Subgroup analysis in this study found that combined exercise significantly improved femoral neck and whole-body BMD. Common forms of combined exercise include resistance training combined with aerobic exercise. Studies by Friedlander et al. (1995) and Batrakoulis et al. (2021) utilized resistance training combined with aerobic exercise at intensities above 70% HRmax and significantly improved femoral neck and whole-body BMD in premenopausal women. Similarly, a meta-analysis by Xiaoya et al. (2025) indicated that combined aerobic and resistance training was the most effective exercise modality for improving femoral neck BMD in postmenopausal women. Mechanistically, resistance exercise applies mechanical forces to the bone, causing interstitial fluid to flow through the bone canaliculi, generating shear stress and membrane deformation at the osteocyte level, thus activating osteocytes and initiating the bone formation process (Robling et al., 2002; Sherk and Rosen, 2019). In addition, resistance training can indirectly promote osteogenesis through muscle contractions that release myokines (such as irisin and IGF-1) (Miao et al., 2025). Recent research has also highlighted the role of the “musculoskeletal axis,” where exercise induces the secretion of OC in bones, activating muscle IL-6 release. IL-6 then circulates through the bloodstream and returns to the bones, binding to the upregulated IL-6 receptor, directly promoting osteoblast function (Palmisano et al., 2023). Some studies have found that after a single session of high-intensity interval training, serum IL-6 levels show a positive correlation with bone formation marker P1NP, without enhancing bone resorption (Sasimontonkul and Sirivarasai, 2024). Aerobic exercise, on the other hand, improves nutrient delivery, induces the release of myokines such as irisin, enhances osteocyte survival (Sherk and Rosen, 2019), and helps improve mitochondrial function, thereby delaying cellular aging (Crane et al., 2010). The World Health Organization (WHO) recommends that adults engage in both aerobic and muscle-strengthening activities, and combining various exercise types may provide additional benefits for bone health (Bull et al., 2020).

In addition to combined aerobic and resistance exercise, resistance training combined with whole-body vibration (WBV) is also an effective approach. WBV provides high-frequency mechanical stimulation via a vibration platform, which, when combined with resistance training, enhances mechanical input to the bones (Rauch et al., 2010). A study by Zinner et al. (2017) found that combined resistance training and WBV significantly promoted cortical bone formation in the femur in young adults. The effectiveness of WBV is influenced by factors such as vibration frequency, amplitude, and participant posture (Kiiski et al., 2008). Humphries et al. (2009) used progressive WBV combined with resistance training at 50 Hz frequency and 1–6 mm amplitude. After 16 weeks of intervention, they significantly improved femoral neck BMD in young women. Additionally, team sports such as soccer have been included in the category of combined exercise. Soccer involves high-intensity, multidirectional movements such as sprinting, changing directions, and tackling. The high acceleration and impact load generated by these actions effectively stimulate bone accumulation (Vainionpää et al., 2007). Research by Krustrup et al. (2010) showed that twice-weekly, 1-h small-sided soccer training significantly increased participants’ whole-body BMD (+1.3%) and lower limb muscle strength after 16 months. Several studies suggest that before engaging in high-intensity impact exercises, strength training or low-impact activities should be performed to enhance muscle strength and endurance, thereby reducing the risk of injury and promoting bone adaptation more effectively (Rodrigues et al., 2021). This progressive, composite exercise strategy helps optimize bone structure and improve BMD.

### 4.2 Effects of exercise on bone turnover markers in adults

After adulthood, the skeleton continues to undergo remodeling, a process that involves a dynamic balance between bone resorption and formation (Owen and Reilly, 2018). Bone turnover markers (BTMs) reflect the activity of osteoblasts and osteoclasts. Osteoclasts secrete acidic substances and specific enzymes (e.g., cathepsin K) during bone resorption, degrading the bone matrix and releasing type I collagen fragments (such as CTX and NTX) as well as TRACP-5b, which serve as biochemical markers for bone resorption (Eastell and Szulc, 2017). The subsequent bone formation phase is dominated by osteoblasts, which synthesize bone matrix and promote its mineralization, releasing substances like OC, P1NP, and BALP, which act as markers of bone formation (Schini et al., 2023).

Studies by Hinton et al. (2015) found that 12 weeks of resistance and jumping exercise significantly increased OC levels but had no significant effect on CTX or TRACP. Similarly, Prawiradilaga et al. (2020) reported that a single high-intensity jumping exercise session significantly increased P1NP and OC levels, with no significant changes in CTX. Taken together, these findings suggest that the positive effects of exercise on bone density are likely due to the promotion of bone formation rather than the inhibition of bone resorption. Mechanistically, the “mechanostat” hypothesis supports this concept: when mechanical strain sensed by osteoblasts exceeds a certain threshold, it activates their proliferation and bone-forming activity (Frost, 2003; Scott et al., 2008). This process involves the transduction of mechanical signals into biochemical signals within the cells, subsequently initiating bone remodeling pathways (Zernicke et al., 2006; Lombardi et al., 2019). Additionally, bone turnover markers themselves are influenced by various factors. For example, CTX levels exhibit circadian fluctuations and are modulated by nutritional status (Qvist et al., 2002), which adds complexity to the interpretation of results. Exercise type is also a key factor. Dolan et al. (2020a) noted that low-intensity repetitive loading activities (e.g., cycling) have a more pronounced effect on CTX than high-intensity or resistance training. Most of the bone resorption-related studies included in this analysis involved high-load or high-frequency exercises (e.g., high-impact exercises, resistance vibration training), which may have influenced the response of bone resorption markers.

Regarding intervention period, exercise interventions shorter than 4 months were more likely to elevate OC and P1NP levels, while BALP levels increased significantly regardless of whether the intervention was shorter or longer than 4 months. For example, Davidović Cvetko et al. (2022) found that 8 weeks of progressively increased interval aerobic exercise significantly raised serum OC levels in young adults. In terms of the bone remodeling timeline, mechanical stimulation typically induces a 3-week osteoclast-dominated resorption phase, followed by a 3-month osteoblast-mediated formation phase (Dolan et al., 2020b; Dolan et al., 2022). This may explain why short-term interventions significantly increased OC, P1NP, and BALP. Additionally, BALP levels continued to rise during long-term interventions, reflecting the sustained osteoblast activity due to ongoing mechanical stimulation, thereby promoting a positive feedback loop for bone formation.

This meta-analysis has several potential limitations that need to be acknowledged. First, the number of studies included in some subgroups was small, which may have reduced statistical power and impacted the detection of significant effects. Second, due to the predominance of studies focusing on adult women, there were fewer studies involving men, and no further subgroup analysis by gender was conducted. As a result, we were unable to fully elucidate potential gender differences in the effects of exercise interventions. Furthermore, there was considerable heterogeneity in the specific parameters of exercise interventions (e.g., intensity, frequency, and type) across the studies, which may have influenced the interpretation of the combined results. Future research should include more high-quality, rigorously designed studies to further clarify the impact of exercise on bone health.

## 5 Conclusion

This systematic review and meta-analysis indicate that exercise significantly increases lumbar spine and femoral neck BMD in young adult women under the age of 30. Additionally, combined exercise shows significant benefits in improving femoral neck and whole-body BMD in adult women. Furthermore, in terms of bone metabolism, exercise effectively promotes the elevation of bone formation markers OC and BALP in adults. Specifically, short-term interventions (less than 4 months) significantly increase OC and P1NP levels, while BALP levels show significant increases following both short-term and long-term (≥4 months) interventions.

## Data Availability

The original contributions presented in the study are included in the article/Supplementary Material, further inquiries can be directed to the corresponding author.
