# Supplementary material for: Effects of exercise on bone mineral density and bone turnover markers in adults: a systematic review and meta-analysis
Source: Front Physiol. 2026 Jan 8;16:1672997. doi: 10.3389/fphys.2025.1672997 (PMC12823962; doi:10.3389/fphys.2025.1672997)
Supplement: Supplementary file 1 [file Supplementaryfile1.docx]

LS

FN

WB

OC

BALP

P1NP

TRACP-5b

CTX
